# The CEDAR Study: A Longitudinal Study of the Clinical Effects of Conventional DMARDs and Biologic DMARDs in Australian Rheumatology Practice

**DOI:** 10.1155/2017/1201450

**Published:** 2017-05-23

**Authors:** Lynden Roberts, Kathleen Tymms, Julien de Jager, Geoffrey Littlejohn, Hedley Griffiths, Dave Nicholls, Paul Bird, Jennifer Young, Julie Hill, Jane Zochling

**Affiliations:** ^1^Department of Medicine, Monash University, Clayton, VIC, Australia; ^2^Rheumatology Department, Australian National University, Canberra, ACT, Australia; ^3^Pacific Private Clinic, Southport, QLD, Australia; ^4^Barwon Rheumatology Service, Geelong, VIC, Australia; ^5^Coast Joint Care and University of the Sunshine Coast, Sunshine Coast, QLD, Australia; ^6^University of New South Wales, Sydney, NSW, Australia; ^7^Roche Products Pty Limited, Sydney, NSW, Australia; ^8^McCloud Consulting Group, Sydney, NSW, Australia; ^9^Menzies Institute for Medical Research, Hobart, TAS, Australia

## Abstract

**Objectives:**

To observe the choices of conventional disease modifying antirheumatic drugs (cDMARDs) and biologic DMARDs (bDMARDs) in the management of rheumatoid arthritis (RA) in Australian routine clinical practice, to assess treatment survival and determine the effect of cDMARDs/bDMARDs on disease activity.

**Methods:**

Routinely collected, deidentified clinical data was sourced from 20 Australian rheumatology practices. RA patients aged ≥18 years, who had received cDMARDs/bDMARDs and a recorded subsequent visit, were included. A linear mixed model was used to determine the change over time and the percentage reduction in disease activity was summarized.

**Results:**

12,526 RA patients were included: 72% females, mean age 62 years. cDMARDs and bDMARDs were used in 92% and 30% of patients, respectively. The most commonly prescribed cDMARD was methotrexate (76% patients); median time to stopping treatment was 337 months [95% CI: 279–ND]. Etanercept was the most commonly prescribed bDMARD (12% patients); median time to stopping treatment was 79 months [95% CI: 57–93]. Of 5,341 patients with a first change in medication (cDMARD or bDMARD), 87% had therapy escalation and 13% deescalation. Reduction in DAS28-ESR, 6-month post-DMARDs initiation ranged from 3%, adalimumab, to 14%, leflunomide and tocilizumab.

**Conclusions:**

In this large Australian cohort of unselected community RA patients, the choices of cDMARDs/bDMARDs are aligned with current international guidelines.

## 1. Introduction

High-quality clinical research underpins our knowledge of the efficacy of various rheumatoid arthritis (RA) therapies [1–4]. Randomized clinical trials (RCTs) have provided reassuring safety and efficacy evidence to support the use of the numerous new therapeutic options for RA over the last 20 years [1–3]. However, in real world practice, outside of the controlled environment of these RCTs, additional factors are likely to influence the use of conventional disease modifying antirheumatic drugs (cDMARDs) and biologic DMARDs (bDMARDs).

RCTs are conducted on carefully selected groups of patients under strict clinical conditions; therefore, it is unclear how the efficacy and safety signals observed in the RCTs will translate into the uncontrolled, relatively unselected environment of routine clinical practice.

There are limited routine clinical practice data available that assess the uptake of new treatments in RA patients [5, 6]. Such data would be useful to better understand any barriers to the implementation of new evidence and would be likely to inform strategies designed to enhance rational prescribing.

The OPAL-QUMI (Optimising Patient outcomes in Australian rheumatoLogy-Quality Use of Medicines Initiative) consortium [7] is a point of care-derived observational registry database with clinical data recorded by Australian rheumatologists in real-time during routine clinical encounters. Cross-sectional data from OPAL has helped to provide insights into the reasons why a proportion of RA patients remain in moderate disease activity using a treat-to-target strategy [8].

## 2. Methods

### 2.1. Study Design

CEDAR is a multicentre, longitudinal, observational study. The primary objective of the study was to identify the choices of cDMARDs/bDMARDs being made over time for the management of RA in Australian patients. The secondary objectives were to assess the survival of RA patients on treatment and the effects of cDMARDs/bDMARDs on disease activity.

The study which was approved by the University of New South Wales Human Research Ethics Committee (reference number HC12094) was conducted in accordance with local guidelines, as set out in the National Statement on Ethical Conduct in Human Research.

### 2.2. Inclusion Criteria

Patients diagnosed with RA, aged ≥ 18 years, who had received any medication, including cDMARDs, bDMARDs, corticosteroids, or NSAIDs, either as monotherapy or in addition to their stable previous therapy, and who also had subsequent follow-up visits as well as a change in cDMARDs/bDMARDs recorded were eligible to be included in the study. Patients who requested their data not to be collected for research purposes were excluded from the analyses.

### 2.3. Data Capture

The study was conducted by the OPAL-QUMI using the clinical software program Audit4 (described previously) [7]. Deidentified data was sourced from 20 participating rheumatology practices comprising 42 rheumatologists across Australia. Data was captured in May 2012. For the assessment of treatment effect on disease activity, the baseline data was collected in May 2012 and 6-month data was collected in November 2012.

### 2.4. Clinical Observations

Parameters collected included demographics (gender and age), diagnosis (clinical onset of RA and disease duration), disease measures (including DAS28-ESR), tender joint count (TJC), swollen joint count (SJC), and laboratory observations (inflammatory markers, autoantibody status, RF and/or ACPA positivity) and medications (NSAIDs, steroids, cDMARDs and bDMARDs with initiation dates, duration of treatment, and reason for cessation).

However, being point of care clinical data, there are parameters missing for some analyses (e.g., effect of treatment on disease activity) in these cases; we have highlighted/defined the number of patients included.

### 2.5. Analytical Assessments and Statistical Considerations

Medication change was analyzed in 9 prespecified categories: methotrexate monotherapy, combination of >1 cDMARDs, combination of methotrexate and leflunomide, monotherapy of cDMARDs other than methotrexate, monotherapy of bDMARDs, combination of bDMARDs plus methotrexate, combination of bDMARDs plus cDMARDs other than methotrexate, combination of bDMARDs and >1 cDMARDs, and no cDMARDs/bDMARDs therapy. Treatment escalation was defined as a new cDMARDs/bDMARDs being introduced and treatment deescalation was defined as the cessation of cDMARDs/bDMARDs.

Treatment survival was defined as the number of months from the first prescription of the drug until the last known date taking the drug. Survival on treatment was analyzed for the most frequently recorded cDMARDs (methotrexate, hydroxychloroquine, leflunomide, and sulfasalazine) and bDMARDs (etanercept, adalimumab, tocilizumab, and abatacept). The number of patients on other biologics was too small for any meaningful analyses to be carried out. Government subsidized bDMARDs sequentially became available in Australia for etanercept in 2003, adalimumab in 2004, abatacept in 2008, and tocilizumab in 2010. Hence, data for tocilizumab, in particular, was over a shorter timeframe in this study. The EULAR response criteria were defined according to van Gestel et al., 1996 [9].

Descriptive statistics [mean, standard deviation (SD), and range] were provided for continuous variables and frequency counts for categorical variables. The median and distribution of treatment survival were estimated using Kaplan-Meier methodology. A linear mixed model was used to determine change over time and all medications with adequate sample size were included. The assumptions of the linear mixed model were that the observations are normally distributed with a constant variance and independence. The assumptions were assessed graphically.

## 3. Results

### 3.1. Patients

A total of 12,526 RA patients were screened: 72% females, mean age 62 years (range 18–100 years). Demographics and disease characteristics for the bDMARDs and bDMARDs groups are presented in Table 1. There were 57% of patients with a baseline DAS28-ESR measurement and the mean score was 3.2 (SD 1.56). RF and ACPA positivity rate was not available for the study; diagnosis of rheumatoid arthritis was made by the treating rheumatologist.

### 3.2. Prescribed Medications

Ninety-two percent (*n* = 11,511) of patients had at least one cDMARD, 30% (*n* = 3,697) had at least one bDMARD, 58% (*n* =7,223) had an oral corticosteroid, and 30% (*n* = 3,699) had at least one NSAID (mainly meloxicam and celecoxib). The most commonly prescribed cDMARDs and bDMARDs were methotrexate (75.9%; *n* = 9,508) and etanercept (12.3%; *n* = 1,545), respectively, with all prescribed cDMARDs and bDMARDs presented in Table 2.

### 3.3. Treatment Survival

**Figure 1:**
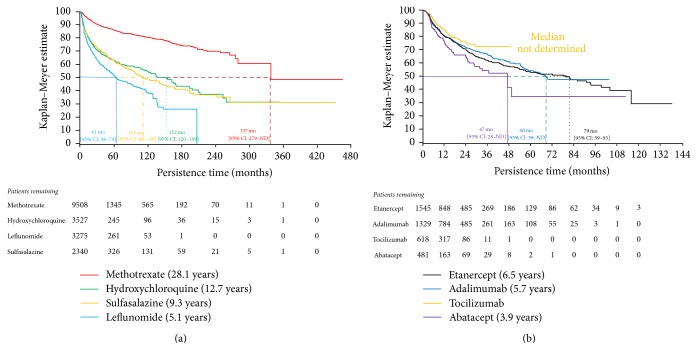
Kaplan-Meier plot of treatment survival: (a) cDMARDs and (b) bDMARDs. ND = not determined.

The treatment survival curves are presented in Figures 1(a) and 1(b). The median time to stopping treatment was 337 months [95% CI: 279–ND] for methotrexate, 152 months [95% CI: 120–168] for hydroxychloroquine, 112 months [95% CI: 96–140] for sulfasalazine, and 61 months [95% CI: 58–79] for leflunomide, while, for patients receiving bDMARDs, the median time to stopping treatment was 79 months [95% CI: 57–93] for etanercept, 68 months [95% CI: 58–ND] for adalimumab, and 47 months [95% CI: 28–ND] for abatacept. The median time to stopping tocilizumab was not reached at the time of data analysis. For patients commencing cDMARDs, Kaplan-Meier (KM) estimates show that 95% [95% CI: 94.5–95.5] of the patients who commenced on methotrexate, 82% [95% CI: 80.7–83.6] on hydroxychloroquine, 77% [95% CI: 75.0–78.2] on leflunomide, and 82% [95% CI: 80.1–83.7] on sulfasalazine persisted on the treatment at 12 months.

Similarly for bDMARDs, KM estimates show that 80% [95% CI: 77.9–82.4] of patients who commenced on etanercept, 81% [95% CI: 78.2–82.9] on adalimumab, 85% [95% CI: 81.5–88.0] on tocilizumab, and 75% [95% CI: 70.0–80.1] on abatacept persisted on the treatment at 12 months.

### 3.4. Medication Choice over Time

**Figure 2:**
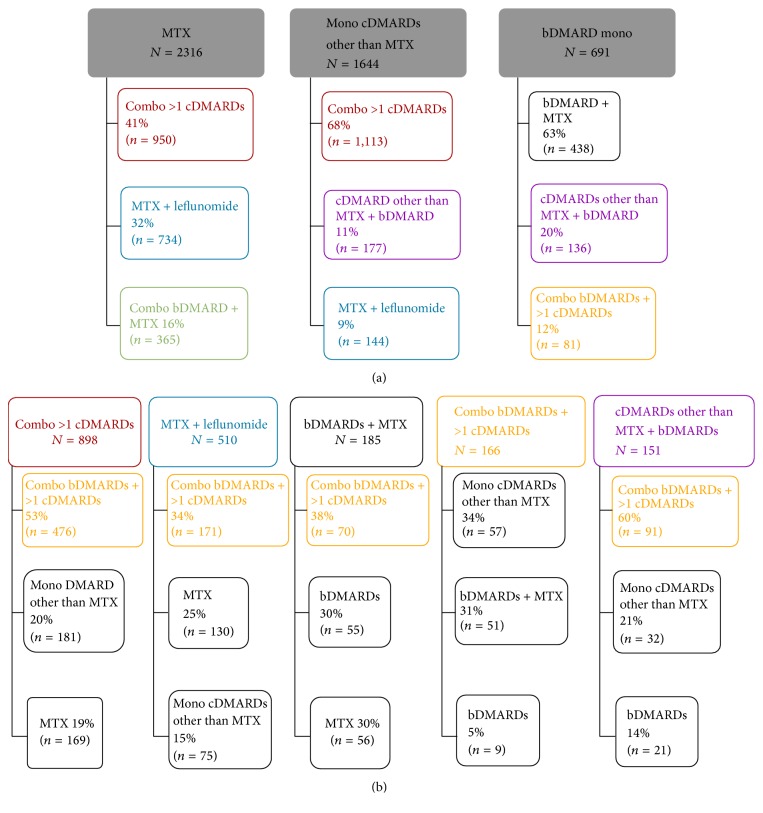
Most common treatment changes in RA medications at first and second change. (a) Treatment algorithm observed when DMARDs were first changed. (b) Treatment algorithm observed when DMARDs were changed a second time. Boxed groups at the top of each figure represent the patient's medication group prior to the medication change. The lines lead to boxes beneath that represent the distribution of medication groups after the medication change. Combo = combination; mono = monotherapy; MTX = methotrexate. Percentages may not add up to 100% as only the most common changes are included.

A total of 5,341 patients had a first change in medication after receiving their initial RA medication. Of these, 4,660 (87%) had their treatment escalated and 681 (13%) had their treatment deescalated. The most common first therapeutic RA medication changes were treatment escalations from monotherapy methotrexate, monotherapy cDMARDs other than methotrexate, and monotherapy bDMARDs (Figure 2(a)).

Two thousand one hundred and eighty-four patients had a second change in their medication; 1,244 (57%) had their treatment escalated and 940 (43%) had their treatment deescalated. The most common treatment changes in RA medications at a second change are shown in Figure 2(b), which illustrates five of the most common decisions (combination of more than one cDMARD, methotrexate plus leflunomide, bDMARDs plus methotrexate, combination of bDMARDs plus more than one cDMARD, and cDMARDs other than methotrexate plus bDMARDs).

### 3.5. Disease Activity

Over a 6-month period, the change in disease activity was assessed following the commencement of a DMARD. Statistically significant, although clinically modest, reductions in mean DAS28-ESR were observed for methotrexate (9.4%), hydroxychloroquine (9.6%), leflunomide (13.8%), sulfasalazine (12.9%), etanercept (8.9%), and tocilizumab (13.9%). A reduction in DAS28-ESR (3.4%) was also observed with adalimumab; however, this was not statistically significant (Table 3).

EULAR responses at 6 months were analyzed for 9 medications (as combination or monotherapy): methotrexate, hydroxychloroquine, leflunomide, sulfasalazine, etanercept, adalimumab, tocilizumab, abatacept, and rituximab, with the remaining medications not having sufficient data to record a EULAR response (Table 4).

## 4. Discussion

This study describes the use of cDMARDs/bDMARDs and medication survival in a large cohort of unselected community RA patients. Conventional DMARD usage reflected the contemporary treatment paradigm with methotrexate, hydroxychloroquine, leflunomide, and sulfasalazine being the predominant cDMARDs used. Over one-quarter of patients in this study used leflunomide, which is a higher rate of usage than what has been observed in Europe and North America [10, 11]. This may be because leflunomide is one of the cDMARD options listed for use prior to a patient accessing government funded biologics in Australia. For bDMARDs, almost 30% of Australian RA patients had been exposed to at least one bDMARD with etanercept, adalimumab, tocilizumab, and abatacept being the most common.

Treatment survival was long for all the studied cDMARDs. Methotrexate had the longest treatment survival and appeared to be fulfilling a role as an anchor medication, as it was also the most commonly used medication. The median time on treatment was shorter for bDMARDs compared to cDMARDs. This may be because, in Australia, patients must fail to respond to at least 2 cDMARDs prior to accessibility to government funded bDMARDs. Accordingly, the patient group receiving bDMARDs in Australia are selected as nonresponders and therefore may represent a group with more refractory disease compared to the unselected group of patients receiving cDMARDs. It is to be expected that the response rates in this more refractory group might therefore be reduced. Nevertheless, the bDMARD median survival rates were similar to those seen in an Italian registry cohort [12].

Australian rheumatologists appear to closely follow the EULAR treatment guidelines for use of medications in the treatment of RA [4]. At the first medication change in patient treatment, the most common escalation pathway was from monotherapy methotrexate. The most common second medication change was the addition of a bDMARD to the combination cDMARDs regimens. These pathways are generally consistent with both the NICE and ACR guidelines updated in 2013 and 2012, respectively [13, 14], with the exception that, in OPAL patients, initial monotherapy cDMARD treatment is considerably more common than initial combination therapy.

The modest reductions in DAS28-ESR over 6 months for the bDMARDs in the OPAL patient cohort appear to be lower than what might have been expected based on previously reported RCTs [15–18] and other observational studies [5, 6, 19–22]. There are a number of reasons why this might be the case. For the RCTs the patient population was selected with a high disease activity, while the published observational studies had stricter selection criteria for the patients in their analyses. In our study the average bDMARD DAS28-ESR prior to medication changes was 3.4, which is considerably lower than the average DAS28-ESR seen in RCTs (often around 6.0) and observational registries (around 5.1–6.0) [5, 6, 15–21] using the same medications. A higher starting DAS28 provides the opportunity for a larger fall in DAS28 following the commencement of a medication, and this may explain the differences between the OPAL and other reported bDMARD responses. We were unable to explain the observed lower baseline DAS28-ESR for patients starting bDMARDS; presumably the treating rheumatologist considered there was sufficient disease activity to warrant commencement of biologic treatment.

There are limitations in the ability to make comparisons between drugs in this study. Rather than being randomly allocated, medications were selected based on individual patient comorbidities and clinician perceptions of different medication efficacy/risk profiles. In addition, Australian government rules, which restrict funding support for bDMARDs until there is a documented failure to respond to at least 2 cDMARDs, may also play a role [2]. Because these and other variables are not able to be controlled in this study design, any outcome differences might be explained by differences in, for example, disease severity/refractoriness being unequally allocated in the treatment groups.

In conclusion, this longitudinal study in a large cohort of unselected community-based RA patients from OPAL clinics identified that medication choices in the Australian rheumatology clinics were in keeping with international guidelines. One exception was that there appeared to be less use of initial combination cDMARDs therapy. Treatment survival was long in all DMARDs groups studied. The first RA medication changes were escalations from monotherapy methotrexate, cDMARDs other than methotrexate, and bDMARDs. Improvements in disease activity were seen in all DMARDs studied. Initiation of cDMARDs/bDMARDs was associated with disease activity reduction 6 months after commencement of treatment.

## Figures and Tables

**Table 1:** Patient demographics and disease characteristics.

	*n*	Mean	SD	Min–max
Age (years)	12,526	61.9	14.0	18.0–100.0
cDMARDs^#^	11,511	61.8	13.9	18.0–99.0
bDMARDs^*∗*^	3,697	59.0	13.1	19.0–91.0
Disease duration (years)	9,069	11.1	10.6	0–77.4
cDMARDs	8,591	11.1	10.6	0–77.4
bDMARDs	2,387	13.7	10.5	0.3–61.1
DAS28-ESR	7,112	3.2	1.6	0–8.8
cDMARDs	6,728	3.2	1.6	0–8.8
bDMARD**s**	2,576	3.4	1.7	0–8.7
Tender joint count	8,842	3.6	5.6	0–28.0
cDMARDs	8,285	3.6	5.5	0–28.0
bDMARDs	3,057	4.4	6.3	0–28.0
Swollen joint count	8,842	3.6	5.5	0–28.0
cDMARDs	8,285	3.7	5.5	0–28.0
bDMARDs	3,057	4.2	6.1	0–28.0
Rheumatoid factor	4,115	137.4	297.5	0–5088.0
cDMARDs	3,846	139.4	300.0	0–5088.0
bDMARDs	1221	158.9	344.1	0–4886.0
ACPA	2282	110.5	198.3	0–2000.0
cDMARDs	2137	110.5	193.0	0–2000.0
bDMARDs	726	124.7	210.4	0–1816.0

SD = standard deviation; ^#^patients taking at least one cDMARD; ^*∗*^patients taking at least one bDMARD.

**Table 2:** Distribution of treatments in the total patient cohort (*N* = 12,526). Multiple occurrences of the same medication in one individual were counted once.

cDMARDs	*n* (%)	bDMARDs	*n* (%)
Patients with at least one treatment	11,511 (91.9%)	Patients with at least one treatment	3,697 (29.5%)

Methotrexate	9,508 (75.9%)	Etanercept	1,545 (12.3%)
Hydroxychloroquine	3,527 (28.2%)	Adalimumab	1,329 (10.6%)
Leflunomide	3,275 (26.1%)	Tocilizumab	618 (4.9%)
Sulfasalazine	2,340 (18.7%)	Abatacept	481 (3.8%)
Sodium aurothiomalate	114 (0.9%)	Golimumab	376 (3.0%)
Azathioprine	98 (0.8%)	Rituximab	339 (2.7%)
Cyclosporine	89 (0.7%)	Certolizumab pegol	273 (2.2%)
Auranofin	43 (0.3%)	Infliximab	68 (0.5%)
Penicillamine	28 (0.2%)	Anakinra	3 (<1.0%)
Cyclophosphamide	10 (0.1%)		

**Table 3:** Change in DAS28-ESR scores over a 6-month period.

Treatment as monotherapy or in combination	*n*	Month 0 mean (SEM)	Month 6 mean (SEM)	Change over time value (%)	*P* value
Methotrexate	1,850	3.19 (0.03)	2.89 (0.03)	0.30 (9.4%)	<0.001
Hydroxychloroquine	494	3.33 (0.06)	3.01 (0.06)	0.32 (9.6%)	<0.001
Leflunomide	472	3.53 (0.07)	3.04 (0.06)	0.49 (13.8%)	<0.001
Sulfasalazine	319	3.55 (0.09)	3.09 (0.08)	0.46 (12.9%)	<0.001

Adalimumab	324	2.96 (0.07)	2.86 (0.06)	0.10 (3.4%)	0.106
Etanercept	394	3.15 (0.07)	2.87 (0.06)	0.28 (8.9%)	<0.001
Tocilizumab	128	2.51 (0.112)	2.16 (0.10)	0.35 (13.9%)	0.001

**Table 4:** EULAR responses.

EULAR response *n* (%)	Methotrexate	Hydroxychloroquine	Leflunomide	Sulfasalazine	Etanercept	Adalimumab	Tocilizumab	Abatacept	Rituximab
Good	279 (15.1%)	77 (15.6%)	81 (17.2%)	57 (17.9%)	46 (11.7%)	33 (10.2%)	19 (14.8%)	7 (12.3%)	9 (13.2%)
Moderate	322 (17.4%)	106 (21.5%)	113 (23.9%)	50 (15.7%)	72 (18.3%)	53 (16.4%)	21 (16.4%)	10 (17.5%)	8 (11.8%)
No response	1,249 (67.5%)	311 (63.0%)	278 (58.9%)	212 (66.5%)	276 (70.1%)	238 (73.5%)	88 (68.8%)	40 (70.2%)	51 (75.0%)
